# Tasmanian Aborigines step up to health: evaluation of a cardiopulmonary rehabilitation and secondary prevention program

**DOI:** 10.1186/1472-6963-14-349

**Published:** 2014-08-18

**Authors:** Maureen Davey, Wendy Moore, Julia Walters

**Affiliations:** Tasmanian Aboriginal Centre, GPO Box 569, Hobart, 7001 Australia; School of Medicine, Medical Sciences Precinct, University of Tasmania, 17 Liverpool St, Hobart, 7000 Australia; Breathe Well: Centre of Research Excellence for Chronic Respiratory Disease and Lung Ageing, Medical Sciences Precinct, University of Tasmania, 17 Liverpool St, Hobart, 7000 Australia

**Keywords:** Aboriginal, Indigenous, Tasmania, Cardiac rehabilitation, Pulmonary rehabilitation, Health service access

## Abstract

**Background:**

Although the burden of cardiopulmonary diseases in the Aboriginal community is high, utilisation of rehabilitation services has been poor. We evaluated the uptake and effectiveness of a cardiovascular and pulmonary rehabilitation program specifically designed and provided for the Aboriginal community, by the Tasmanian Aboriginal Centre, for people with diagnosed chronic heart or respiratory disease and those at high risk of developing such conditions.

**Methods:**

Participants had established chronic obstructive pulmonary disease, ischaemic heart disease or chronic heart failure or were at high risk of developing such diseases because of multiple risk factors. Rehabilitation programs (n = 13) comprised two exercise and one education session per week over eight weeks. Data, collected at baseline and on completion, included health status, risk factors, attendance, anthropometric measurements, physical capacity and quality of life. Data from participants who attended at least one program session were analysed. Qualitative written feedback from participants and staff was analysed thematically.

**Results:**

Of 92 participants (39% with an established disease diagnosis), 72 provided follow-up data. Participants lost weight, and waist circumference decreased (mean -3.6 cm, 95% confidence interval (CI)-2.5 to -4.7). There were clinically significant improvements in six-minute walk distance (mean 55.7 m, 95% CI 37.8 to 73.7) and incremental shuttle walk (mean 106.2 m, 95% CI 79.1 to 133.2). There were clinically significant improvements in generic quality of life domains, dyspnoea and fatigue. Generally, the improvements in participants with established cardiac or respiratory diseases did not differ from that in people with risk factors. Analysis of qualitative data identified three factors that facilitated participation: support from peers and health workers, provision of transport and the program structure. Participants’ awareness of improvements in their health contributed to ongoing participation and positive health outcomes, and participants would recommend the program to family and friends.

**Conclusion:**

A cardiopulmonary program, which included exercise and education and met national guidelines, was designed and delivered specifically for the Aboriginal community. It increased participation in rehabilitation by Aborigines with, or at high risk of, established disease and led to positive changes in health behaviours, functional exercise capacity and health related quality of life.

## Background

Chronic cardiovascular and respiratory diseases are common causes of disease burden and premature mortality in the Aboriginal community [1]. These types of diseases often co-exist in the same person as they share many underlying social determinants and risk factors including poverty, underemployment, poor housing, racism, smoking, obesity, poor nutrition and physical inactivity, which all have a much higher prevalence in the Aboriginal population [2].

Cardiac and pulmonary rehabilitation programs generally have positive effects for participants including improved quality of life and physical capacity [3, 4] and reduced mortality and hospital admissions [5]. Exercise training in a combined program has been shown to be effective in increasing exercise tolerance, functional status and health status in patients with Chronic Obstructive Pulmonary disease (COPD) and Chronic Heart Failure (CHF) [3]. Nationally the availability, uptake and adherence to rehabilitation programs are below recommended levels [6] while uptake is even less for Aborigines [7–9]. There are no published data on utilisation of cardiac and pulmonary rehabilitation services by Aborigines in Tasmania, but workers in both Aboriginal health and rehabilitation sectors report almost no participation. Recent initiatives include training healthcare practitioners to deliver culturally appropriate pulmonary rehabilitation to Aborigines [10]. A cardiac rehabilitation program structured and delivered by an urban Aboriginal Medical Service was well attended and reduced cardiovascular risk factors [11]. However, there remains a need for accessible and culturally appropriate cardiopulmonary rehabilitation services to address the high burden of disease and premature mortality associated with chronic cardiovascular and pulmonary diseases in the Aboriginal community. Furthermore, interventions needed for primary and secondary prevention involve activities similar to those in programs designed for rehabilitation. Indeed, the *Australian National Preventive Health Strategy 2009 road map for action* recommends focusing on reducing risk factors, especially in socially and economically disadvantaged populations [12].

In the face of very low utilisation of existing cardiac and pulmonary rehabilitation services by Aborigines with cardiovascular or respiratory disease in Tasmania, a project to deliver cardiopulmonary rehabilitation was established in October 2011 by the Tasmanian Aboriginal Centre (TAC), an Aboriginal community-controlled organisation with a 20-year history of providing comprehensive primary health care services to Aborigines. To address the additional aim of secondary prevention, the program was open to those who had at least two risk factors, in addition to Aboriginality, for developing cardiovascular disease.

The hypotheses were that the provision of culturally accessible cardiac and pulmonary rehabilitation would increase participation and improve health outcomes for people with established disease, and would reduce risk factors such as obesity and physical inactivity for people with risk factors.

We report our findings on participation and effectiveness of a combined cardiovascular and pulmonary rehabilitation and secondary prevention program provided by an Aboriginal community controlled health service.

## Methods

The program design was based on the principles and experience of Aboriginal community-controlled health services which have initiated and continue to provide comprehensive primary health care services to Aborigines while addressing the deep-rooted social, political and economic conditions that prevail in those communities [13]. Guidelines from the National Heart Foundation and Lung Foundation of Australia were used to ensure consistency with nationally accepted best practice in cardiac and pulmonary rehabilitation [8, 14, 15].

The programs were situated within the TAC and integrated with its other clinical and health promotion programs. The goal was to create an ongoing sustainable program that was of direct benefit to participants and promoted the benefits of physical activity to other Aboriginal health service programs and the wider Aboriginal community. Participant inclusion criteria were diagnosis of chronic obstructive pulmonary disease (COPD), ischaemic heart disease (IHD) or heart failure (CHF), and people with at least two cardiovascular risk factors (smoking, obesity, hypertension, diabetes, dyslipidaemia).

The decision to include people with cardiac disease and pulmonary disease in the same program as each other, and with people with multiple risk factors, was based on the holistic, integrated and comprehensive approach of the Aboriginal community controlled health sector [13]. Many potential program participants had dual diagnoses, or a single diagnosis and risk factors for the other disease, or multiple risk factors for both chronic cardiovascular and respiratory disease. The educational program was designed to be inclusive of cardiovascular and pulmonary health and disease, and the exercise programs were individually prescribed, monitored and reviewed.

The program design and implementation involved extensive collaboration with public and private sector physiotherapists and exercise physiologists. Aboriginal Health Workers (AHWs), who have formal clinical and health promotion training, played a key role in recruiting and supporting participants, and liaising between the Aboriginal Health Service and external health professionals.

The programs were of eight weeks duration, consisting of two supervised exercise sessions of one hour (16 sessions total) and one educational session (8 sessions total) lasting at least one hour per week. The programs commenced in Launceston and Hobart in October 2011, and, although the recruitment and assessment processes were similar in both places, there were some differences in the implementation, which are described below.

The educational sessions were facilitated, interactive and accompanied by a healthy lunch at the TAC. The sessions promoted self-management approaches and covered cardiovascular and respiratory health and disease; benefits of exercise; shopping, cooking and eating healthy food; medication usage; stress and psychological well-being; and smoking cessation.

Individual baseline assessments were used to classify level of risk and measure fitness including identification of maximal load. These assessments were used to calculate a safe but challenging level of exercise. During the program exercise intensity was monitored with subjective scales for shortness of breath and rate of perceived exertion. Heart rates could not be used as a reliable measure of intensity as many participants were taking medication. The exercise physiologists reviewed and upgraded the individual exercise prescriptions on a weekly basis. Participants set and modified goals throughout the program often using the information gained from initial testing.

In Hobart the physical activities included use of stationary bicycles, steps and weights, but in Launceston the program incorporated outside walking as well as this gym equipment. All participants were encouraged to participate in physical activities on other days of the week.

In Launceston the exercise and educational programs were delivered at the TAC, and provided by an experienced, hospital-based cardiac rehabilitation physiotherapist. The programs were adapted and implemented in consultation with the TAC. From January 2013, the same program was delivered by an outreach exercise physiologist from a private physiotherapy practice. All participants attended the twice-weekly exercise and once-weekly education sessions as a group.

In Hobart, the exercise programs were prescribed and monitored by an exercise physiologist based in a local private physiotherapy practice; timing of sessions was flexible to suit individual participants, especially those in employment. The program participants were paired up for twice-weekly exercise sessions in the gym of the physiotherapy practice. In January 2013 the exercise sessions moved to a new room at the TAC with the exercise physiologist from the same private practice attending on an outreach basis. The weekly educational sessions were held at the TAC, coordinated by the TAC AHW, and delivered by the TAC doctors, nurses and dietician, as well as the exercise physiologist.

All staff had training in first aid, access to basic first aid equipment including oxygen, and access to phones to call other staff and an ambulance. Transport was provided for participants who would otherwise have had difficulty attending.

The TAC provided management, professional and logistical support on a statewide basis. The programs were supported by government funding through Medical Specialist Outreach Assistance Program – Indigenous Chronic Disease (MSOAP-ICD) and the core funding of the TAC health service.

### Recruitment

Potential participants were adult Aborigines identified by the AHW through the use of the chronic disease and risk registers of the TAC patient information management system (Communicare) and discussion with the clinical AHWs, nurses and doctors. As well as people referred through these clinical processes, some participants referred themselves because of the positive stories told by family and friends who had completed earlier programs.

The selection of participants for a particular program was intentional, not sequential or random. Priority was given to people with established cardiac and/or chronic respiratory disease, or those at high risk due to the presence of risk factors including diabetes, long-term smoking, hypertension, dyslipidaemia and obesity. Of potential participants eight people were selected for each program with the intention to recruit equal numbers of men and women for each group. Staff knowledge of the local Aboriginal families and community was used to deliberately recruit people who would have the active support, and be supportive, of at least one other participant in that particular group.

Potential participants were approached by the AHW to assess interest and ability to commit to participating in the intensive eight-week program. Participants gained a medical clearance from a TAC general practitioner who took note of the standard exclusion criteria for exercise-based rehabilitation (myocardial infarction, unstable angina and certain arrhythmias). Cardiology consultation was sought for prospective participants with complex or severe cardiovascular disease.

Participants commenced programs simultaneously in eight-week blocks. The paired or grouped exercise setting was designed to support motivation and ongoing participation. On completion, participants were encouraged to attend ongoing supervised maintenance exercise.

### Outcome measures

Measurements were obtained during the week before and after the program by the physiotherapist or exercise physiologist with an AHW.

Data collected included age, gender, health conditions, reason for referral, risk factors, body mass index and waist circumference, and attendance at exercise and education sessions. Physical capacity assessment was conducted according to Australian rehabilitation guidelines [14, 15] and measured by the Six Minute Walk Test (6MWT), clinically important change ≥35 metres in COPD [16] and > 45 metres in heart failure [17], and the Incremental Shuttle Walk Test (ISWT), clinically important improvement ≥ 79 metres in COPD [18] and improvement of 80–100 metres in cardiac rehabilitation [19]. Functional capacity was assessed using the Timed Up and Go test (TUG) of lower limb function and mobility [20, 21]. We used the scales for dyspnoea and fatigue in the Chronic Respiratory Questionnaire (CRQ) [22] which are identical in the Chronic Heart Failure Questionnaire [23] and relate to symptoms of breathlessness for personally selected activities and non-specific fatigue. The threshold for clinical improvement in the CRQ is 0.5 [24]. Quality of life was measured by the generic Medical Outcomes Short Form (SF36) [25]. On completion of the program all participants were invited to submit an evaluation form which included questions on motivation to take part and persist in rehabilitation, health and wellbeing changes due to rehabilitation, program content and satisfaction, and also provided space for free-text responses. Staff were invited to participate in semi-structured interviews and written feedback.

### Ethics

The evaluation was a joint project of the Tasmanian Aboriginal Centre and the UTAS School of Medicine Centre for Research Excellence for Chronic Respiratory Disease and Lung Ageing. Ethics approval was granted by the State Committee of the Tasmanian Aboriginal Centre and the Human Research Ethics Committee Tasmania Network (Ref No: H0012041). Informed consent in writing was obtained from all participants prior to participation. The trial was retrospectively registered with ANZCTR on 7 August 2014 with ACTRN12614000842662.

### Analysis

All Aborigines who completed the baseline assessment and attended at least one exercise session in a program held between October 2011 and July 2013 were included in the evaluation sample. Quantitative analysis of participation and rehabilitation outcomes was performed on STATA, version 12. Demographic and clinical characteristics of participants with or without established cardio-respiratory disease were compared at baseline using chi-squared and t-tests. Paired t-tests were used to compare outcome measures pre- and post-rehabilitation and Cohen’s d used to calculate effect sizes for all participants, and separately for those with or without established cardio-respiratory disease. A p-value of less than 0.05 was considered statistically significant and no adjustment was made for multiple comparisons [26]. Qualitative thematic analysis of feedback from participants and staff was undertaken by one researcher (MD) to identify, code and collate themes through an iterative process where the themes and sub-themes were reviewed and changed throughout the process. The two other authors read transcripts to verify and discuss themes. Microsoft Word 2007 was used to store, code, segment and amalgamate evaluation data.

## Results

### Description of participants

Between October 2011 and February 2013 there were 13 programs involving 92 participants who contributed data. A diagnosis of established disease, IHD or CHF or COPD was present for 36 (39%) participants, 11 of whom also had diabetes. There were 56 participants without a diagnosis of cardiovascular disease or COPD, but with two or more risk factors; 50% had dyslipidaemia, 32% had diabetes, 35% were current smokers, 60% had hypertension and 76% were obese (BMI > 30). A large proportion of participants (n = 69, 75%) had significant and long-term health conditions such as arthritis, depression, schizophrenia, post-traumatic stress disorders, and alcohol or other substance abuse issues, as well as their cardiac or respiratory condition and risk.

The intention to recruit equal numbers of men and women was not fully achieved with 61% female participants overall. When grouped by established disease versus risk factors only, proportions were similar by gender and employment status (Table 1), although those with established disease were significantly older (Table 2) than those with risk factors only and more were current smokers (64% versus 36%). Both groups had a high rate of obesity (BMI >30), 49% in established disease and 77% in participants with risk factors. Only 6% of participants in both groups had waist circumference measurements below the threshold recommended by the Heart Foundation in Australia. Participants with risk factors had significantly higher weight and BMI at baseline than participants with established disease, however there were no significant differences in Incremental Shuttle Walk Test, Six Minute Walk Test, generic quality of life (SF36), or CRQ dyspnoea and fatigue (Table 2). Baseline anthropometric, physical capacity, mobility and quality of life measures did not differ for participants who did not attend for follow-up, with the exception of lower weight and smaller waist circumference in non-attenders (data not shown).

**Table 1:** **Baseline characteristics of participants enrolled in TAC rehabilitation program**

	All n = 92	%	CCRD ^1^ n = 36	%	RF ^2^ n = 56	%
Age group^†^						
<=49	35	38.0	7	19.4	28	50.0
50–59	35	38.0	17	47.2	18	32.1
> = 60	22	23.9	12	33.3	10	17.9
Sex	n = 80					
Female	56	60.9	21	58.3	35	62.5
Smoking*						
Never	20	21.7	2	5.6	18	32.1
Ex-smoker	28	30.4	11	30.6	17	30.4
Current	43	46.7	23	63.9	20	35.7
BMI status						
Normal <25	11	12.2	8	22.9	3	5.5
Overweight 25–30	20	22.2	10	28.6	10	18.2
Obese >30	59	65.6	17	48.6	42	76.4
Employment status						
Not in workforce	59	64.1	22	61.1	37	66.1
Employed P/T	13	14.1	4	11.1	9	16.1
Employed F/T	19	20.7	9	25.0	10	17.9
Recorded diagnosis (n = 92)						
COPD^3^			20	55.6		
IHD^4^			21	58.3		
CHF^5^			6	16.7		
Hypertension	57	62.0	23	63.9	34	60.7
Dyslipidaemia^	57	62.0	29	80.6	28	50.0
Diabetes	29	31.5	11	30.6	18	32.1
Other condition	69	75.0	26	72.2	43	76.8

Footnotes: ^1^CCRD = Chronic Cardiovascular and Respiratory Disease, ^2^RF = risk factor only, ^3^COPD = Chronic Obstructive Pulmonary disease, ^4^IHD = Ischaemic Heart Disease, ^5^CHF = Chronic Heart Failure.

^†^CCRD v RF Pearson chi2 = 8.8823, P = 0.01 *CCRD v RF Pearson chi2 = 11.4902, P = 0.01, ^CCRD v RF Pearson chi2 = 8.6799, Pr = 0.01.

**Table 2:** **Baseline anthropometric, physical capacity, mobility and quality of life measurements for participants attending follow up**

	All participants n = 72	CCRD ^1^ n = 25	RF ^2^ n = 47
Age^†^	50.7 (11.3)	56.4 (9.1)	47.6 (11.3)
Weight (kg)*	95.6 (23.5)	86.7 (19.4)	100.5 (24.2)
BMI^	34.6 (7.9)	31.5 (6.4)	36.2 (8.3)
Waist (cm)	111.6 (16.6)	108.9 (15.5)	113.1 (17.1)
ISWT^3^ (m)	557.4 (243.1)	551.1 (203.3)	561.3 (268.5)
6MWT^4^ (m)	438.8 (132.9)	450.0 (125.6)	432.9 (137.6)
TUG^5^ (sec)	5.5 (2.1)	5.7 (1.7)	5.3 (2.3)
SF36^6^ (0–100 good)			
Physical function	66.1 (22.4)	60.6 (24.7)	69.6 (20.5)
Role physical	60.2 (40.0)	58.7 (41.0)	61.1 (39.8)
General health	50.6 (23.3)	47.9 (23.1)	52.2 (23.5)
Bodily pain	59.0 (28.0)	63.0 (26.0)	56.5 (29.2)
Vitality	48.2 (22.8)	46.0 (22.0)	49.5 (23.4)
Social functioning	74.5 (27.0)	72.9 (29.1)	75.5 (26.0)
Role emotional	66.1 (42.8)	66.7 (41.5)	61.2 (21.8)
Mental health	61.6 (22.8)	62.2 (24.7)	61.2 (21.8)
CRQ^7^ (0–7 best)			
Dyspnoea	4.1 (1.7)	3.8 (2.0)	4.2 (1.5)
Fatigue	4.0 (1.6)	4.3 (1.3)	3.8 (1.8)

Results presented as mean (standard deviation).

Footnotes: ^1^CCRD = Chronic Cardiovascular and Respiratory Disease, ^2^RF = risk factor only, ^3^ISWT = Incremental Shuttle Walk Test, ^4^6MWD = Six Minute Walk Test, ^5^TUG = Timed Up & Go, ^6^SF36 = Medical Outcomes Short Form, ^7^CRQ = Chronic Respiratory Questionnaire.

^†^CCRD v RF ttest t = -3.3432, P = 0.001, *CCRD v RF ttest t =2.4441, P = 0.02, ^CCRD v RF ttest t =2.4515, P = 0.02.

### Participation

Exercise attendance ranged from 1–16 sessions, mean 11.5 sessions, SD 3.1; however, 79% of participants attended at least half. Educational attendance ranged from 0–8 sessions, mean 5.4 sessions, SD 2.4; 70% of participants attended at least half. Participants with cardiovascular disease and COPD attended fewer exercise sessions (mean difference -3.5, 95% CI -1.9 to -5.0) and fewer education sessions (mean difference -1.2, 95% CI -0.2 to -2.2) than participants with risk factors only. To facilitate attendance, transport to the program was provided if required; 48% of participants always used transport and 15% sometimes. The rate of use did not differ according to the presence of established disease or risk factors only. Attendance at exercise sessions was similar for the programs in Hobart and Launceston and was not affected by age and gender or employment status. Reasons for non-completion included acute medical problems such as myocardial infarction, cardiac arrhythmias, exacerbation of COPD, and family and work responsibilities. Following completion of the program, similar proportions of participants continued attending maintenance exercise sessions: 25% with established disease and 36% with risk factors (p = 0.3).

### Changes following the TAC program

Outcome data were obtained at follow-up for 72 (78%) participants, 25 with CHF, IHD or COPD (69%) and 47 with risk factors only (84%). Demographics and baseline clinical measures were similar for those who did or did not attend for follow-up, with the exception of lower weight and smaller waist circumference in non-attenders.

Following the program, there were significant improvements in anthropometric outcomes among participants overall: weight (effect size 0.04), waist circumference (effect size 0.22) and BMI (effect size 0.04) (Table 3). Improvements were similar in established disease and risk factors only groups, although with small sample sizes in each group they did not achieve statistical significance.

**Table 3:** **Changes in anthropomorphic measurements and physical capacity after rehabilitation for a) all participants, b) participants with cardiac and/or chronic respiratory disease (CCRD), c) participants with risk factors (RF)**

	All participants			CCRD			RF		
	n	Mean (95% CI)	ES	n	Mean (95% CI)	ES	n	Mean (95% CI)	ES
Weight (kg)	64	-0.8 (-0.1, -1.6)	0.04	21	-1.0 (-2.5, 0.4)	0.01	43	-0.8 (-1.7, 0.2)	0.04
BMI^1^	64	-0.3 (-0.01, -0.6)	0.04	21	-0.3 (-0.6, 0.1)	0.02	43	-0.3 (-0.8, 0.2)	0.03
Waist (cm)	66	-3.6 (-2.5, -4.7)	0.22	23	-4.9 (-6.8, -2.9)	0.08	43	-3.0 (-4.3, -1.7)	0.17
ISWT^2^ (m)	47	106.2 (79.1, 133.2)	0.11	19	96.8 (40.3, 153.4)	0.12	28	112.5 (84.5, 140.5)	0.10
6MWT^3^ (m)	64	55.7 (37.8, 73.7)	0.11	22	79.0 (47.1, 111.0)	0.17	42	43.6 (21.9, 65.2)	0.10
TUG^4^ (sec)	67	-0.8 (-0.5, -1.1)	0.11	23	-0.8 (-1.3, -0.3)	0.14	44	-0.8 (-1.1, -0.5)	0.10

Data shown as mean change from baseline, 95% confidence intervals (CI) and Effect Size (ES).

Footnotes: ^1^BMI = Body mass index, ^2^ISWT = Incremental Shuttle Walk Test, ^3^6MWD = Six Minute Walk Test, ^4^TUG = Timed Up & Go.

There were improvements among participants overall in exercise capacity, measured as the Incremental Shuttle Walk Test (effect size 0.11) and Six Minute Walk Test (effect size 0.11) (Table 3); the mean improvement in all participants and those with COPD and cardiovascular disease exceeded the minimal clinically important differences. The Timed Up & Go test at baseline was better than published data in community dwelling older adults [27] although an improvement was seen after the program (effect size 0.11). Changes of similar magnitude for these outcomes occurred in the established disease group and the risk factors group (Table 3).

Quality of life improved in all domains of the SF36 for participants overall; changes were statistically significant in the general health, bodily pain, vitality social functioning, role emotional and mental health domains (Table 4). Although the magnitude of the change that reflects an important improvement in SF36 has not been determined, changes seen correspond to small to medium clinical improvements in COPD and heart disease [25]. In the established cardiac and respiratory disease group vitality, role emotional and mental health domains showed statistically significant improvement. Improvements of similar magnitude occurred in the risk factors only group: statistically significant for role physical, general health, bodily pain, vitality and mental health domains.

**Table 4:** **Changes in quality of life after rehabilitation for a) all participants, b) participants with cardiac and/or chronic respiratory disease (CCRD), c) participants with risk factors (RF)**

	All participants		CCRD		RF	
	n	Mean (95% CI)	n	Mean (95% CI)	n	Mean (95% CI)
SF 36^1^ (0–100 good)						
Physical function	61	5.1 (-0.9, 11.1)	24	6.3 (-2.1, 14.6)	37	4.3 (-4.3, 12.9)
Role physical	58	11.2 (-0.5, 22.9)	23	5.4 (-14.7, 25.5)	35	15.0 (0.1, 29.9)
General health	60	9.7 (4.4, 14.9)	22	3.7 (-4.5, 12.0)	36	13.3 (6.5, 20.1)
Bodily pain	58	7.4 (0.5, 14.4)	23	4.9 (-8.0, 17.8)	37	9.3 (0.5, 17.5)
Vitality	59	15.3 (9.6, 21.1)	24	12.1 (0.7, 23.5)	35	17.6 (11.5, 23.7)
Social functioning	59	8.5 (0.8, 16.3)	23	8.7 (-4.5, 22.0)	36	8.4 (-1.7, 18.5)
Role emotional	59	13.5 (1.0, 26.1)	23	16.0 (1.6, 30.3)	36	12.0 (-6.9, 30.9)
Mental health	59	14.2 (8.6, 19.9)	24	14.3 (7.2, 21.4)	35	14.2 (5.6, 22.7)
CRQ^2^ (0–7 best)						
Dyspnoea	45	0.6 (0.1, 1.0)	20	0.9 (0.2, 1.5)	25	0.3 (-0.3, 0.9)
Fatigue	46	0.8 (0.4, 1.3)	19	0.4 (-0.4, 1.1)	27	1.2 (0.6, 1.7)

Data shown as mean change from baseline, 95% confidence intervals (CI).

^1^SF36 = Medical Outcomes Short Form, ^2^CRQ = Chronic Respiratory Questionnaire.

The CRQ dyspnoea and fatigue scores for all participants improved significantly (Table 4) and changes exceeded the minimal clinically important level; improvements in participants with established cardiac/respiratory disease was statistically significant for dyspnoea. In the risk factor group participants, improvement in fatigue was statistically significant and above the minimal clinically important difference.

### Qualitative results

#### Participant evaluation

The feedback from the 51 participants who completed evaluation forms was very positive, and identified many factors that supported ongoing participation. The most commonly identified reason was receiving encouragement and support from other participants, exercise physiologists, physiotherapists, Aboriginal health workers and other staff at the Aboriginal Health Service. Several identified the importance of the small group and team environment involving other people they already knew as members of the Aboriginal community, “my own people”. Other enablers were the provision of transport, having a structured program, access to equipment and a variety of exercise, feeling healthier, having fun, and learning new information about health.

All participants said they would recommend the program to family and friends without any major changes being required: “Just as it is run now” and “Hard to improve something this good and beneficial” were comments made. Increasing the variety of exercise such as swimming, bike riding, Zumba® and yoga was suggested.

The overwhelming majority of participants (97%) noted improvement in one or more aspects of health and wellbeing on the program; for example feeling more motivated, fitter, more relaxed and sleeping better. A striking proportion commented on the nutritional impact of their changed diet, and weight loss, in addition to being more physically active, getting stronger, walking more easily. Changes in smoking behaviour, smoking less (n = 5), quitting (n = 3) and increasing motivation for making a quit attempt were reported.

#### Staff evaluation

Major themes to emerge from the staff were the benefits of working in a program where they could witness the positive improvements to the health of participants.

They enjoyed “seeing people achieve positive attitudes for themselves, become optimistic about their health’s future”, and “seeing community members who have been involved with this program leading the way with their exercising and healthy attitudes”. Many identified that program participants became more involved in other aspects of the Aboriginal Health Service such as clinic, cooking, quit smoking and exercise maintenance programs.

The AHWs appreciated being able to make a difference, “seeing the change in a small amount of time”, “rewarding to make changes to people’s lifestyle, in particular those who have complex health problems”, and “seeing how healthy people are getting”. They liked being able to concentrate on one program, being part of a multidisciplinary team and learning new skills from the exercise physiologists and physiotherapists, and getting “to know that there are some ways we can beat the onset of chronic illness and disease”. They also appreciated the community interaction, “being part of getting your community helping themselves”, and the “comradeship that develops between participants and staff throughout the program whilst leading to better health for all”.

The non-Aboriginal exercise physiologists appreciated gaining cultural insight into Aboriginal community, feeling valued by participants, and seeing people learn a range of skills and improve levels of function.

The strengths of the program were related to it being designed specifically for Aborigines, being in a familiar environment with motivated and encouraging staff and participants, with a variety of educational topics, a focus on results, and an individualised structured approach to goals and exercise.

“This program and any other program that educates, empowers Aboriginal people to gain beneficial results regarding their health is nothing but a positive for community and families. Let there be more.” (AHW).

## Discussion

We have shown that a well-designed and culturally appropriate cardiopulmonary rehabilitation program had good participation rates by Aborigines in Tasmania and achieved clinically relevant improvements in health outcomes, consistent with our hypotheses. Participants with established cardiac or chronic respiratory disease experienced improvements in several aspects of health. The benefits for weight loss, lower BMI and waist circumference corresponded to small effect sizes, however weight loss and nutritional changes are a goal of both pulmonary and cardiac rehabilitation but are recognised as difficult to achieve [15]. The positive changes in this program may be due to the practical emphasis on nutrition in the TAC program which included provision of information by a nutritionist, the weekly sharing of healthy lunches during the educational sessions, and supermarket visits to check affordable and healthy food options. There were also benefits on quality of life with decreased impact of breathlessness, and improved vitality and emotional and mental health and increased physical capacity. These improvements were comparable on the same outcome measures to those seen in a meta-analysis of pulmonary rehabilitation in COPD [28]. A welcome finding was that participation in the same program led to improvements of similar magnitude for people at high risk of cardiac or chronic respiratory disease, but who had not yet developed actual disease. This program, with its dual focus on rehabilitation and prevention, has achieved its objective to improve health in the Aboriginal population, and fits the recommendations in the *State of Preventive Health 2013* report for intervention efforts that need to be appropriate to specific needs with an enabling infrastructure supported by strong workforce and leadership [29].

The success of the TAC program illustrates that, with the supply of appropriate and accessible services, Aborigines are willing to act to improve their health. The conceptualisation of access at the interface of health systems and populations developed by Levesque, Harris and Russell [30] provides a framework for analysing what enabled participation in the TAC program. The Levesque framework characterises five dimensions of access on the supply side for health services as approachability, acceptability, availability and accommodation, affordability, appropriateness. The ability to utilise effective health interventions depends on the corresponding abilities in the population to perceive, seek, reach, pay and engage. For example, the close connection between the cardiopulmonary program and the established comprehensive primary health care services provided by this Aboriginal community-controlled health service was fundamental to its success. The capacity of the TAC health services to successfully meet supply side dimensions of the Levesque framework have evolved over many years and are ensured by organisational governance, management, staffing and service delivery processes which are answerable to the local Aboriginal community and to funding bodies. Participation in the cardiopulmonary programs was enabled by utilising existing networks of Aborigines and the health care providers who worked in the hosting Aboriginal community-controlled health organisation. The identification and perception of health care needs by potential participants, was supported by Aboriginal Health Workers, nurses, doctors and managers who the promoted the likely benefit of engaging with the cardiopulmonary program, and supported the referral and enrolment process. The co-location of the program with the Aboriginal Health Service facilitated access, as participants were already familiar with the building and staff, and had the added benefit that many participants became more engaged with ongoing clinical, health promotion and community activities run by the TAC. Affordability was maximised by the absence of fees, the provision of transport if needed, the flexibility of exercise time in Hobart to fit in with work and other commitments, and lack of any requirement for specialised clothing or equipment.

The sustainability of the program over time is also associated with it being hosted by an Aboriginal community-controlled health service. The situation is similar to that of the ongoing heart health program hosted by Derbarl Yerrigan Aboriginal Health Service in Western Australia [11]. Ongoing funding, leadership, management and staffing underpin sustained programs. Some of the ways in which the TAC supported the transition from pilot project to an ongoing program included initiating planning and implementation before the availability of specific funding, and continuing support between annual funding rounds. The TAC health promotion coordinator, regional managers and public health medical officer provided ongoing leadership, management and administrative support, and the organisation could provide alternative AHWs if staff were absent because of sickness, holidays or resignation. The existing processes of the TAC provided the clinical and organisational governance for the cardiopulmonary program.

As well as the direct benefit to participants, the program has also created demand in the Aboriginal community for other physical activity, cooking and healthy eating programs, which are now run by the TAC for people who do not meet the disease or risk factor criteria for the cardiopulmonary program.

We recognise that our results are limited by small numbers in subgroups with established cardiac/respiratory disease or risk factors only. This may have reduced the power to detect statistically significant changes for some outcomes, although the direction and magnitude of improvements and effect sizes were similar. Another potential limitation is incomplete outcome ascertainment for physiological and quality of life measures due to withdrawals, which may have biased the findings. Lack of long-term follow-up data do not allow us to see if the improvements were maintained. The integration of the program with other health promotion and community activities supports participants to persist with positive health behaviour changes, with attendance at maintenance exercise programs also likely to favour long-term improvements. We do not have data on the cost-effectiveness of the program although this could be collected in future research.

## Conclusion

A cardiopulmonary program, that included exercise and education and met national rehabilitation program guidelines, was designed and delivered specifically for the Aboriginal community by an Aboriginal community controlled health service. It increased participation in rehabilitation by Aborigines with, or at high risk of, established disease and led to positive changes in health behaviours, functional exercise capacity and health related quality of life.

## Author’s information

MD is a public health physician and general practitioner who is employed as the Public Health Medical Officer at the Tasmanian Aboriginal Centre and as a senior lecturer in public and population health at School of Medicine, University of Tasmania.

WM is a palawa woman who is employed as the State Health Promotion Coordinator at the Tasmanian Aboriginal Centre.

JW is a Senior Research Fellow in Primary Health Care at 'Breathe Well' Centre of Research Excellence for Chronic Respiratory Disease in the School of Medicine, University of Tasmania. She is supported by an NHMRC Primary Health Care Training Fellowship No: 544943.

## Abbreviations

AHWs: Aboriginal health workers
BMI: Body mass index
CCRD: Chronic cardiac and respiratory disease
CHF: Chronic heart failure
CI: Confidence interval
COPD: Chronic obstructive pulmonary disease
CRDQ: Chronic respiratory disease questionnaire
IHD: Ischaemic Heart Disease
ISWT: Incremental shuttle walk test
MSOAP-ICD: Medical Specialist Outreach Assistance Program – Indigenous Chronic Disease
SD: Standard deviation
SF36: Medical outcomes short form
6MWT: Six minute walk test
TAC: Tasmanian Aboriginal Centre
TUG: Timed Up and Go
UTAS: University of Tasmania.
